# Disruptive behavior among elementary students in physical education

**DOI:** 10.1186/s40064-016-2764-6

**Published:** 2016-07-22

**Authors:** José López Jiménez, Alfonso Valero-Valenzuela, M. Teresa Anguera, Arturo Díaz Suárez

**Affiliations:** 1 Faculty of Sports Sciences, University of Murcia, Murcia, Spain; 2 Faculty of Psychology, University of Barcelona, Barcelona, Spain

**Keywords:** Physical education, Elementary school, Disruptive behaviors, Pattern and disinterest

## Abstract

The aim of this study was to determine which disruptive behaviors occur most often in physical education (PE) classes, and to identify the existence of a pattern of behavior that leads to this disruptive behavior. With this in mind, we analyzed five PE sessions taken by pupils at different elementary school in the region of Murcia. The total sample size was 96 students aged between 10 and 13. Data was recorded using an observation instrument (a combination of a field format and a categorical system) and was then analyzed using the “HOISAN” software tool, with a sequential analysis and polar coordinates being conducted. The results of the study revealed that disruptive behaviors (52 %) occur more frequently than non-relevant behaviors (48 %), the most common of them being disinterested behavior (29 %), followed by indiscipline (15 %), with no statistically significant differences being detected in violent behavior. As regards patterns of behavior, disinterested behavior is stimulated by “no eye contact”, “middle distance”, “inside the task”, “no use of material”, “giving orders” and “registering of activities”, while indiscipline is stimulated by “no eye contact”, “far distance”, “outside the task”, “use of material”, “grouping in pairs” and “preparation of material”. In conclusion, it can be stated that disruptiveness is far more common in physical education sessions, affects the development of sessions and has a negative impact on student learning. A solution to this problem should therefore be sought immediately in order to ensure quality education.

## Background

Ensuring quality in the student teaching/learning process is one of the main objectives towards which teachers strive. A positive atmosphere in the classroom is essential to achieving quality and enabling the teacher to create suitable conditions for the teaching of students. However, disruptive behavior in the classroom is playing an increasingly negative role and is occurring with greater frequency (Sulbarán and León 2014). As a result, it now poses one of the biggest and most worrying problems in education (Peña and Ángulo 2015), given that discipline is regarded as one of the most important aspects of teaching and one of the most difficult to deal with in schools (Moreno et al. 2007). It is also one of the most important indicators for most teachers, given that a well-behaved class has a very good chance of being taught successfully (Gotzens et al. 2003; quoted in Gutiérrez et al. 2009).

The issue of discipline in the classroom has become such a problem that teachers are, on occasion, unable to provide a solution to it. As Trianes et al. (2001) rightly point out, this has a major impact on the day-to-day running of schools. Indiscipline can lead to teaching staff becoming demoralized, and can ultimately cause adverse psychological effects, with teachers devoting less time to content on the syllabus, priority objectives being overlooked and less attention being given to children with special requirements.

It is essential to define at this point what is meant by “disruptive behavior”. According to Urbina et al. (2011, p. 3), it is “student behavior that systematically disrupts educational activities, undermines the habitual development of the tasks carried out in the classroom, and causes the teacher to invest a significant amount of time in dealing with it, time that should otherwise be devoted to the processes of teaching and learning.”

According to García (2001), this disruptive behavior has become increasingly prevalent in Europe over the last few decades, the most frequent types of such behavior being the transgression of classroom rules, the interruption of tasks, the challenging of teachers’ authority, and aggression towards other classmates. It is also worth highlighting the categorization provided by Urbina et al. (2011), who identify two types of violence within disruptive behavior. The first of them is verbal violence, which includes insults, shouting, swearing, taunting, jokes, insults and name-calling, all of which are engaged in with the aim of ridiculing, humiliating and unsettling a classmate or teacher or making them feel bad. Secondly, there is physical violence, which includes molesting, manhandling, hitting, kicking and pushing, each with the aim of harassing, attacking or provoking the victim.

The problem of disruptiveness in the classroom, which has become a widespread social problem, has generated no little concern in the scientific community. In response, an increasing number of studies have been published and proposals for intervention put forward (Moreno et al. 2007; Gutiérrez et al. 2009; Latorre and Teruel 2009; Sánchez-Rivas et al. 2015), all with the aim of identifying the origin and cause of this behavior, of analyzing the factors influencing its emergence, and of proposing possible strategies for addressing what is an extremely serious problem (Rodríguez 2011).

A comparison of the disruptive behavior encountered in all subjects on the syllabus reveals that it occurs with similar frequency in each subject (Ishee 2004), even in PE classes, despite the fact they are held in a different location to other classes (Esteban et al. 2012).

In focusing in more detail on PE, which provides the subject of our study, it is vitally important to identify the possible indicators of disruptive behavior so that initial proposals for intervention can be drawn up with a view to eradicating or reducing such behavior in the classroom.

In light of the above, the objectives of this study were to identify what type of disruptive behavior occurs most frequently in PE classes, to analyze which factors relating to the design of the session lead to students behaving disruptively, and, finally, to detail which actions engaged in by the teacher can trigger disruptive behavior.

## Methods

### Design

The observational design approach is nomothetic/punctual (with intrasessional following)/multidimensional (Anguera et al. 2011) Is nomothetic as it enables the disruptive behavior of the students and the behavior of the teacher to be studied; it enables monitoring, as it is carried out during the course of an elementary-school PE session; and it is multi-dimensional, as it is an instrument featuring various response levels.

### Participants

Five video recordings were analyzed in the study. They were made at elementary-school PE sessions at five schools (Conde de Campillos, Federico de Arce, Nuestra Señora de Fátima, Nuestra Señora de los Dolores, and San Pablo) in the region of Murcia. The groups ranged in size between 14 and 25 students, with the study participants ranging in age between 10 and 13. A total of 96 students and five teachers were analyzed. All recordings lasted between 45 and 50 min.

### Instruments

#### Observation instrument

The observation instrument used was a combination of a field format and a category systems (Anguera et al. 2007). The instrument contains a total of 13 response levels, criteria or dimensions, six of which were used to draw up a categorical system (given that there is no theoretical framework and they are timeless), each of them being exhaustive and mutually exclusive. The other seven were used to create a field format, with their respective catalogues, which meet the requirement of mutual exclusivity (Table 1).

**Table 1:** Observation instrument

Level of response/criteria	Categories/index
Type of disruptive behavior	Violence
	Disinterest
	Indiscipline
	Violence + disinterest
	Violence + indiscipline
	Disinterest + indiscipline
	Violence + disinterest + indiscipline
People involved	None
	One person
	Two people
	Between two people and 25 %
	Between 25 and 50 %
	Between 50 and 99 %
	All
Sighting of disruptive behavior	Eye contact
	No eye contact
	Partial eye contact
Distance	Short
	Middle
	Far
	Short + middle
	Short + far
	Middle + far
	Short + middle + far
Student action performed inside the task	Listening to information from the teacher
	Performing the proposed task
Student action performed outside the task	Talking to another classmate
	Performing an activity other than the one proposed by the teacher
	Using the mobile
Material	Yes
	No
Verbal behavior of the teacher inside the task	Information at the start of the task or session
	Giving instructions/orders
	Feedback
	Information at the end of the task or session
Behavior of the teacher outside the task	Talking to another student about aspects unrelated to the session
	Marking work
	Using the mobile or tablet
Reprimand	Telling a student off because they are not doing the proposed task
	Taking a student to task for disruptive behavior directed towards another student or the material
	Telling a student off for cheating
Observational registering of activities	Registering information on the performance of the students
	Observing the performance of students
	Counting up of scores
Preparation/collection material	Preparing the material to be used
	positioning the material in the task area
	Collecting the material used
	Handing out the material to students
Non-relevance	Performing the task proposed by the teacher
	Listening closely to the information given by the teacher
	Observing the rules laid down by the teacher
Non-observation	Technical
	Teacher
	Teacher

A brief explanation of each of the response levels comprised in the observation instrument is provided below:

1. *Type of disruptive behavior* the category of disruptive behavior that arises: disinterest, indiscipline or violence.
2. *People involved in the disruptive behavior* the number of students behaving disruptively.
3. *Sighting of disruptive behavior* the teacher sees the disruptive behavior.
4. *Distance* distance between the teacher and the students behaving disruptively.
5. *Action developing inside the task* the students are partly carrying out the proposed task or listening to the information given by the teacher.
6. *Action developing outside the task* the students are doing anything other than the things they are supposed to be doing “inside the task”.
7. *Equipment* students have some kind of material in their possession when disruptive behavior arises.
8. *Verbal behavior of the teacher inside the task* type of information given by the teacher to students when disruptive behavior occurs.
9. *Behavior of the teacher outside the task* the teacher comments on aspects that are not related to the session, the task, the objectives or the name and/or execution of the task, or is performing other actions that have nothing to do with the session.
10. *Reprimand* the teacher tells off a student or takes them to task for their behavior at a given time or during the session.
11. *Registering/observation of activities* the teacher registers information on the students, watches activities being performed, or joins with them in counting the task scores out loud.
12. *Preparation/collection of material* the teacher prepares the material to be used in carrying out the task, collects the material that has been used, or hands the material out to students.
13. *Non*-*relevance* non-relevant action/behavior that is not pertinent to the aim of the study (as it is not disruptive behavior) and which does not, therefore, prevent the PE class from following its intended course.
14. *Non*-*observation* period of time in which the people that are the subject of the study (students and teacher) are not filmed. Technical non-observation or the non-observation of people occurs when 50 percent of the students are absent from the recording area.

To ensure greater internal consistency of the observation instrument, the intra-rater agreement was calculated by means of Cohen’s kappa (1968) and using the “HOISAN” software tool, with a value of 0.987 being obtained.

#### Recording instrument

- *HDR*-*PJ30VE camcorder* high-definition, hybrid (memory card, 32 GB memory) camcorder. Supports MPG, MTS, AVCHD format, MPEG-2.
- *HOISAN* (Tool for the Observation of Social Interaction in Natural Environments) (Hernández-Mendo et al. 2012): a software tool used to encode, record, describe and manage recordings and to enable real-time viewing from one or more cameras. It can also work with all data types: sequences of events, states, mixed sequences, time intervals and multimodal events. The observational record metrics use primary parameters and derived or secondary measurements. The program has the ability to analyze verbal output and calculate different types of agreement and correlation indices. It also supports data exchange with specific programs for use in observational methodology (SDIS-GSEQ, OBSERVER, THEME and MOTS) (Hernández-Mendo et al. 2014), other general programs (spreadsheets, statistical packages, word processors), and programs for qualitative analysis (Atlas.ti) and the exporting of data to portable document format (PDF).

### Procedure

Following the selection of the schools, a meeting was held with their respective head teachers and PE teachers, who were informed of the purpose of the research study and its duration. The schools were also notified that there would be no need for them to change their timetables. Once the schools had each agreed to the research study being conducted, informed consent forms were handed out to the parents of the students, requesting their permission to record their children on video.

To prevent students from acting unnaturally during the study, a camera was put in place for three sessions prior to the actual recording of the research-study sessions. The PE teachers each placed a camcorder on the sports courts where the sessions were to be held in order to record the behavior occurring during them. The camcorders were positioned in a corner of the sports courts so as not to cause a hindrance during the tasks and to cover as wide an area as possible. Each teacher also set up a wireless microphone to record all their comments on audio during filming.

Once the five recordings of the PE classes had been completed, a bibliographical review was conducted as a means of creating the observation instrument (a combination of the categorical system and the field format). Continual tests were conducted with different videos with a view to adjusting the instrument as far as possible. Inter-rater reliability was then calculated using the kappa coefficient (Cohen 1968).

The five PE sessions were then analyzed using the “HOISAN” software tool, with a sequential analysis of the data and polar coordinates being conducted to enable interpretation of the results.

## Results

The results achieved in this investigation are described below. Firstly, it shows the amount and type of disruptive behavior in elementary-school PE classes through two figures. After it shows the results by means of two data-analysis techniques as the lag sequential analysis and polar coordinates.

**Fig. 1:**
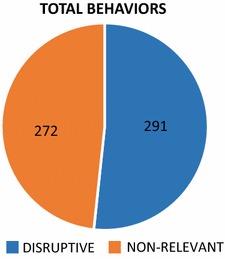
Amount of disruptive and non-relevant behavior occurring in elementary-school PE classes

Figure 1 shows the total amount of behaviors (non-relevant and disruptive) taking place in the PE classes: 560 in all. We divided these behaviors into two major groups: disruptive behaviors and non-relevant behaviors. As Fig. 1 shows, disruptive behaviors occur with greater frequency, in a little over half of all instances (52 %). These behaviors comprise disinterest, indiscipline, violence and a combination of all three, such as disinterest + indiscipline, violence + indiscipline, and violence + disinterest. For its part, non-relevance accounts for slightly less than half of all instances (48 %) and also comprises non-relevant behavior, due to the non-observation of the subjects of the study (students and teacher).

**Fig. 2:**
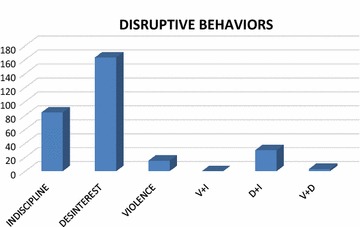
Amount and type of disruptive behavior occurring in the PE classes

In terms of the most frequent type of behaviors occurring in the elementary-school PE classes (Fig. 2), disruptive behavior accounted for 290 behaviors out of the total of 560. The most common type of disruption, and which occurs with the greatest frequency, therefore, is disinterest, which occurs in 29 % of instances, followed by indiscipline (15 %) and the combined behavior of disinterest + indiscipline (5 %). The behaviors occurring with least frequency are violence (2 %), followed by the combined behaviors of violence + disinterest (0.4 %) and violence + indiscipline (0.2 %).

### Lag sequential analysis

One of the data-analysis techniques used was the lag sequential analysis delays, which allows us to identify the existence of patterns of behavior in the categories that form the recording and encoding tool, ahead of the probabilities created by the effect of chance (Barreira et al. 2014; Castellano et al. 2002; Lago and Anguera 2003).

Table 2 shows the results obtained in the sequential analysis of the “disinterest” behavior criterion.

**Table 2:** Patterns of behavior in the five sessions analyzed, with “disinterest” regarded as a behavior criterion

Code	Del−4	Del−3	Del−2	Del−1	Del+1	Del+2	Del+3	Del+4
No eye contact	3.959	−0.323	2.871	−*2.834*	−*2.41*	4.566	−1.806	2.414
Middle distance	3.516	−1.152	4.881	−*2.284*	−*2.979*	3.57	−0.857	3.541
Inside the task	2.722	−0.443	5.188	−1.865	−*3.113*	5.396	−1.898	3.974
No use of material	5.846	−0.34	9.055	−1.062	−0.871	7.586	−0.859	4.732
Whole class	2.668	−*2.358*	3.426	−*2.732*	−*2.732*	2.655	−2.358	1.484
Giving orders	3.052	0.119	2.547	−1.064	−0.822	3.597	0.917	1.388
Registering of activities	2.211	0.039	2.728	−1.011	−0.475	4.068	−0.496	3.507

The underlined figures are the significant excitatory adjusted residuals (positive value higher than 1.96), and the figures in italics are the significant inhibitory adjusted residuals (negative value lower than −1.96), p being considered <0.05

The significant excitatory patterns of behavior detected on the basis of the “disinterest” behavior criterion reveal that immediately before they occur (delay −1), and in a statistically significant manner, the “no eye contact”, “middle distance” and “whole class” behaviors do not occur. However, all conditioned behaviors (“no eye contact”, “middle distance”, “inside the task”, “no use of material”, “whole class”, “giving orders” and “registering of activities”) do have a high probability of occurring, given that the adjusted residuals are all statistically significant and excitatory. Furthermore, and prior to a delay −3 in which only the “whole class” behavior is statistically significant, and in an inhibitory manner, all the behaviors in delay −4 are significantly excitatory once more, with alternation occurring in the delays studied, which is revealing.

Following the occurrence of the “disinterest” behavior criterion, we detect in delay 1 that there is a statistically significant probability that the behaviors (“no eye contact”, “middle distance”, “inside the task” and “whole class”) will not occur, as the adjusted residuals are negative, However, in delay 2 there is a high probability that all the conditioned behaviors (“no eye contact”, “middle distance”, “inside the task”, “no use of material”, “whole class”, “giving orders” and “registering of activities”) will occur, given that the adjusted residuals are all statistically significant and excitatory. In delay 3 only the “whole class” behavior is statistically significant, and in an inhibitory manner, and in delay 4 most of the behaviors, with the exception of “whole class” and “giving orders” are significantly excitatory once more, with alternation also occurring in the delays studied, which is revealing.

This structure should be borne in mind, as it shows the reality prior to disinterest, with a clear and almost complete symmetry between the retrospective and prospective perspective, which backs up the alternation detected. This interpretation leads us to regard disinterest as recurrently and strongly linked to all the behaviors (delays −4, −2, 2 and 4), but in such a way that a negative probability is detected between the successive occurrences of these behaviors (a high probability that it will not occur); i.e. it is inhibitory in nature, in relation to the occurrence of some of said behaviors, as commented above.

Table 3 shows the results obtained in the sequential analysis of the “indiscipline” behavior criterion.

**Table 3:** Adjusted residuals of lag sequential analysis corresponding to the five sessions analyzed, with “indiscipline” being the behavior criterion

Code	Del−4	Del−3	Del−2	Del−1	Del+1	Del+2	Del+3	Del+4
No eye contact	3.377	−0.672	5.031	−1.274	0.078	3.313	1.95	0.857
Far distance	2.248	0.732	4.55	0.32	1.459	3.727	−0.072	1.058
Outside the task	2.01	−0.773	4.828	−0.539	−0.539	4.511	0.596	1.979
Use of material	3.931	2.529	7.835	2.135	1.783	7.398	2.821	3.163
Grouping in pairs	2.858	3.255	4.432	−1.052	−0.277	2.431	2.423	2.416
Preparing of material	2.81	0.548	2.263	0.007	1.675	3.892	2.775	1.105

The underlined figures are the significant excitatory adjusted residuals (positive value higher than 1.96), and the figures in italics are the significant inhibitory adjusted residuals (negative value lower than −1.96), p being considered <0.05

In terms of the patterns of behavior obtained from the “indiscipline” behavior criterion, Table 3 shows that there are no statistically significant behaviors of an inhibitory manner or in the prospective perspective (delay 1, 2, 3 and 4) or the retrospective perspective (delay −1, −2, −3 and −4).

However, in observing the significant excitatory patterns of behavior, we can see how the “use of material” behavior occurs immediately before the indiscipline behavior (delay −1), Furthermore, in delay −2 there is a high probability that the conditioned behaviors (“no eye contact”, “far distance”, “outside the task”, “use of material”, “grouping in pairs”, “preparation of material”) will occur, as they are all statistically significant and excitatory. Moving even further back to delay −3, the only statistically significant and excitatory behaviors are “use of material” and “grouping in pairs”, after which all the behaviors become statistically significant and excitatory again in delay −4, as is also the case in delay −2.

Further data reveals that there is a high probability of all the behaviors (“no eye contact”, “far distance”, “outside the task”, “use of material”, “grouping in pairs”, “preparation of material”) occurring in delay 2, as they are statistically significant and excitatory. In delay 3 only some of the behaviors are statistically significant and excitatory, namely “use of material”, “grouping in pairs” and “preparation of material”, with these last two behaviors also appearing in delay 4, in addition to “outside the task”.

Consequently, in the delays studied, the “indiscipline” behavior criterion does not reveal the same alternation (excitation and inhibition) as the aforementioned “disinterest” behavior criterion. However, “indiscipline” can be linked to a very strong probability of all the behaviors appearing in delays −4, −2, 2 and 4, some of them alternating in the successive occurrences of behavior.

Table 4 shows the results obtained in the analysis of the “disinterest + indiscipline” behavior criterion.

**Table 4:** Patterns of behavior corresponding to the five sessions analyzed, with “disinterest + indiscipline” being the behavior criterion

Code	Del−4	Del−3	Del−2	Del−1	Del+1	Del+2	Del+3	Del+4
Short distance	0.288	2.405	1.354	2.946	5.061	1.882	1.349	0.318
No use of material	1.677	2.841	0.538	4.786	4.4	0.945	0.936	1.721
No grouping	3.078	3.501	1.016	3.518	4.349	2.706	1.866	2.294
Registering of activities	3.103	4.727	0.958	2.582	0.963	−0.661	1.492	0.41

The underlined figures are the significant excitatory adjusted residuals (positive value higher than 1.96), p being considered <0.05

As the table shows, there are no statistically significant inhibitory behaviors in any of the delays studied (−4, −3, −2, −1, 1, 2, 3 and 4).

There are statistically significant and excitatory behaviors, with there being a high probability of all the conditioned behaviors (“short distance”, “no use of material”, “no grouping” and “registering of activities”) occurring immediately before the “indiscipline + disinterest” behavior criterion (delay −1). The same situation arises in delay −3, though this is not the case in delay −4, where only the “no grouping” and “registering of activities” behaviors are statistically significant and excitatory.

With regard to the prospective perspective, we can see how the behaviors “short distance”, “no use of material” and “no grouping” occur just after the “indiscipline + disinterest” behavior criterion (delay 1), after which only “no grouping” occurs (delay 2 and 4), given that the adjusted residual is statistically significant and excitatory.

### Polar coordinates analysis

**Fig. 3:**
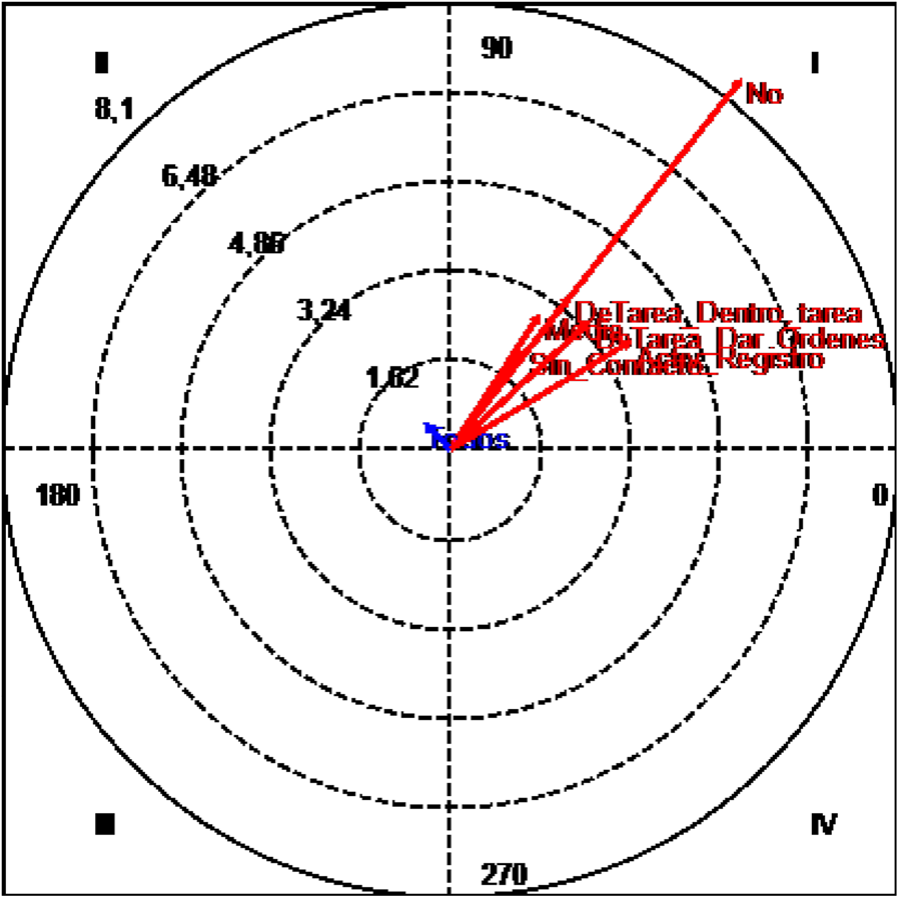
Polar coordinates of the “disinterest” behavior criteria and all the conditioned behaviors

Polar coordinates is another technique used to analyze data and enables us to create a vector representation of the relationships established between the focal behavior (the object of analysis) and the rest of the behaviors that make up the recording and encoding tool (Castañer et al. 2016; Castellano and Hernández-Mendo 2003; Gorospe and Anguera 2000) (Fig. 3).

#### Quadrant 1:

There is a relationship of mutual excitability between the “disinterest” focal behavior and the conditioned behaviors “no eye contact”, “middle distance”, “inside the task”, “no use of material”, “giving orders” and “registering of activities”. This is statistically significant.

#### Quadrant 2:

**Fig. 4:**
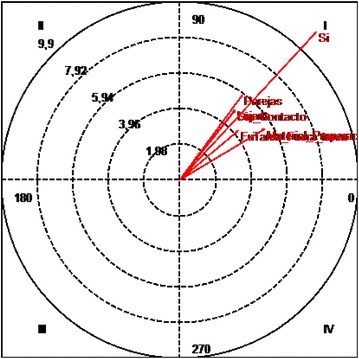
Polar coordinates of the “indiscipline” behavior criteria and all the conditioned behaviors

The “disinterest” focal behavior has an inhibitory effect on the “whole class” conditioned behavior although this conditioned behavior also has an excitatory effect on the “disinterest” focal behavior. This is not statistically significant (Fig. 4).

#### Quadrant 1:

There is a relationship of mutual excitability between the “indiscipline” focal behavior and the conditioned behaviors “no eye contact”, “far distance”, “outside the task”, “use of material”, “grouping in pairs” and “preparation of material”. This is statistically significant.

#### Quadrant 1:

**Fig. 5:**
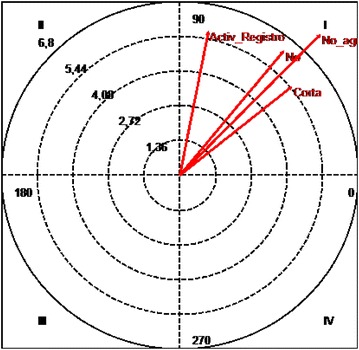
Polar coordinates of the “disinterest + indiscipline” behavior criteria and all the conditioned behaviors

There is a relationship of mutual excitability between the “disinterest + indiscipline” focal behavior and the conditioned behaviors “short distance”, “no use of material”, “no grouping” and “registering of activities”. This is statistically significant (Fig. 5).

## Discussion

This study set out to identify which disruptive behaviors occurred most frequently in PE classes and whether there was a pattern of behaviors relating to the organization of the session itself or the teacher’s actions that led to students behaving disruptively. In view of this, a sequential analysis of delays was carried out with the “HOISAN” software tool, which allowed us to carry out a retrospective and prospective analysis of the sessions, aiding the search for this common pattern of behaviors. Polar coordinates were also calculated as a means of visualizing the degree of excitation and/or inhibition that may exist between focal behaviors (“disinterest”, “indiscipline”, “disinterest + indiscipline”, and “violence”) and all the conditioned behaviors.

In view of the results obtained, it can be said that disruptive behaviors in elementary-school PE classes are slightly more in evidence than non-relevant behaviors; i.e. inappropriate behaviors on the part of students (disinterest, indiscipline, etc.) take up more time than the time devoted to the task itself. Other studies, such as the one conducted by Ramírez and Justicia (2006), also indicate that disruptive behaviors are the most frequent of all behaviors in the classroom. However, the results of the study carried out by Rodríguez (2011), contrast with those obtained in this research work, as there is an absence of conflict in the schools analyzed.

This situation is also highlighted by Gotzens, Badía, Genovard and Dezcallar (2010) in their study, where they state that approximately half the time in the classroom is devoted to activities that have nothing to do with the content to be taught by the teacher and everything to do with problems of discipline. On occasion, this causes a reduction in the learning time of students and a distraction for inexperienced teachers (Esteban, et al. 2012). As we shall see, the appearance of these disruptive behaviors is intimately linked to the design and structure of the session and to the actions of the teacher during it.

It has thus been found that disinterest is the behavior occurring most frequently during PE sessions. Some of the behaviors associated with this type of behavior include talking to other classmates while the teacher is explaining the session or tasks and not paying attention to the explanation given; interrupting the teacher; and interrupting the teacher while they are giving information. The reason for the appearance of this disinterested behavior could lie with the student’s lack of interest and/or motivation towards PE, and in particular to the content of the class, even when there is a high competitive factor. The higher frequency of this disinterested behavior in relation to other behaviors can also be found in the compulsory secondary-school education phase, as detected in previous studies, where “talking” is the most common inappropriate behavior in PE (Esteban et al. 2012). Unlike these authors, we include talking as part of more general behavior, such as disinterest.

Another type of disruptive behavior that arises frequently in PE sessions is indiscipline. As we have seen, this behavior appears in less than a third of the sessions, though it is very prevalent when it does so, and the consequences it can have on the student teaching/learning process are more negative than disinterested behavior for example. This disruptive behavior is thus linked in general terms to a failure to observe the rules set out by the teacher. In more specific terms, these behaviors involve complaining continuously about something that has happened in the session; cheating in performing the tasks proposed; and interrupting other classmates where they are performing a task, whether it is listening to the teacher while they are explaining something or while another classmate is performing a motor task. Other behaviors associated with indiscipline are making improper use of the material available or damaging it, and not following the instructions given by the teacher, i.e. not performing the tasks proposed by the teacher or doing a different task, altering the original design of the activity.

Violent behavior (the other major type of disruptive behavior) was found to appear only infrequently during PE sessions, compared to the two behaviors mentioned above (disinterest and indiscipline). Violent behavior is the most worrying of all due to its seriousness and because the intention on the part of the student in question is to cause harm to other classmates. It is essential, therefore, to find a solution to it immediately. The appearance of this behavior seems to be linked both to verbal attacks, such as insults directed to other classmates, and physical attacks, such as hitting, kicking, pushing or tripping up fellow classmates.

It was also noted that the aforementioned disruptive behaviors appeared in combination with other behaviors, occurring, for example, at the same time as disinterest + indiscipline (being significant in nature) or violence + indiscipline behaviors.

According to the data obtained, all these disruptive behaviors are associated with patterns of behavior that cause a student to be disruptive in PE classes or increase the probability of that happening.

Firstly, student disinterest is linked to a series of behaviors that can increase the probability of it arising. They include the following: when students are inside the task and carrying out the activities proposed by the teacher; when students are not in possession of material; when the organization of the class for the performing of tasks is group-based, i.e. the students perform the activity jointly. As regards aspects relating to the teacher’s actions, there is a link between disinterest and the following circumstances: the teacher does not have eye contact with students behaving in a disinterested manner; there is a middle distance between the students and the teacher; the teacher is watching and/or recording the tasks being performed by students; the teacher joins with the students in counting task scores out loud; the teacher gives instructions to other classmates during the session.

It has also been noted that indiscipline is linked to another significant group of behaviors. As regards the structure of the session, we find that there is a high probability of indiscipline occurring in the following circumstances: when students are outside the task, i.e. when they are performing an activity other than the one proposed by the teacher; when students are in possession of material; and when the task organization is pair-based. Furthermore, in relation to the actions of the teacher, we have identified a significant link between indiscipline and circumstances when the teacher does not have eye contact with the students engaging in said behavior, when the teacher is far away from the students, and when the teacher is preparing, collecting or handing out material.

Finally, the combined behavior of disinterest + indiscipline is triggered when the teacher is at a short distance from the students; when they are not in possession of material; when there are no groupings, i.e. when the activities are performed by students individually; and when the teacher is observing and/or recording the tasks being performed by the students, or joins with them in counting the task scores out loud.

In view of the results obtained and detailed above, it is for this reason that there are many elements that teachers need to take greater care of, both in terms of designing sessions and of their own actions, in order to reduce or eradicate this type of disruptive behavior, which undermines peaceful coexistence in the classroom and which is so detrimental to the student learning process.

As regards the limitations of the research study, we should point to the small number of sessions analyzed (5), which prevented a more stable pattern of behavior from being obtained. A comparison could also be made between the disruptive behavior encountered in primary and secondary education to ascertain if there are differences between one cycle and the other in terms of the amount and type of these disruptive behaviors.

Finally, it would also be worth monitoring several sessions at the same centre and applying one of the teaching approaches to check its effectiveness as a potential solution to the problem.

## Conclusions

- The number of disruptive behaviors in elementary-school PE classes is high, and is slightly higher than non-relevant behaviors. This is a cause for concern, and a solution must be found in order to safeguard quality standards in teaching.
- The most frequent disruptive behavior occurring in PE classes is disinterest, followed by indiscipline and the combined behavior of disinterest + indiscipline. Violent disruptive behavior occurs only in this cycle and does not generate statistically significant data.
- There is a close relationship between some disruptive behaviors and some behaviors relating to the session and the actions of the teacher, which cause disruption or raise the probability of occurring.
- Disinterest is stimulated by “no eye contact”, “middle distance”, “inside the task”, “no use of material”, “giving orders” and “registering of activities”. Indiscipline is stimulated by “no eye contact”, “far distance”, “outside the task”, “use of material”, “grouping in pairs” and “preparation of material”. Finally, disinterest + indiscipline are stimulated by “short distance”, “no use of material”, “no grouping” and “registering of activities”.

## Abbreviations

PE: physical education
PDF: portable document format
