# Erratum to: Disruptive behavior among elementary students in physical education

**DOI:** 10.1186/s40064-016-2989-4

**Published:** 2016-08-18

**Authors:** José López Jiménez, Alfonso Valero-Valenzuela, M. Teresa Anguera, Arturo Díaz Suárez

**Affiliations:** 1 Faculty of Sports Sciences, University of Murcia, Murcia, Spain; 2 Faculty of Psychology, University of Barcelona, Barcelona, Spain

## Erratum to: SpringerPlus (2016) 5:1154 DOI 10.1186/s40064-016-2764-6

Upon publication of the original article (López Jiménez et al. 2016), it was noticed that the Funding information was omitted. This has now been included below in this erratum.

### **Funding**

We gratefully acknowledge the support of the Spanish government project La actividad física y el deporte como potenciadores del estilo de vida saludable: Evaluación del comportamiento deportivo desde metodologías no intrusivas (Secretaría de Estado de Investigación, Desarrollo e Innovación del Ministerio de Economía y Competitividad) during the period 2016-2018 [Grant DEP2015-66069-P].
